# Blood donor screening for West Nile virus (WNV) revealed acute Usutu virus (USUV) infection, Germany, September 2016

**DOI:** 10.2807/1560-7917.ES.2017.22.14.30501

**Published:** 2017-04-06

**Authors:** Daniel Cadar, Philipp Maier, Susanne Müller, Julia Kress, Michael Chudy, Alexandra Bialonski, Alexander Schlaphof, Stephanie Jansen, Hanna Jöst, Egbert Tannich, Stefan Runkel, Walter E Hitzler, Gabriele Hutschenreuter, Martina Wessiepe, Jonas Schmidt-Chanasit

**Affiliations:** 1 Bernhard Nocht Institute for Tropical Medicine, WHO Collaborating Centre for Arbovirus and Hemorrhagic Fever Reference and Research, Hamburg, Germany; 2 German Centre for Infection Research (DZIF), partner site Hamburg-Luebeck-Borstel, Hamburg, Germany; 3 Institute for Transfusion Medicine, University Hospital, Aachen, Germany; 4 Paul-Ehrlich-Institut, Federal Institute for Vaccines and Biomedicines, Langen, Germany; 5 Transfusion Center, University Medical Center of the J.G. University, Mainz, Germany; 6 These authors contributed equally to this work

**Keywords:** Usutu virus, West Nile virus, Blood donor, PCR, Human infection, Germany, outbreak

## Abstract

Between 1 June and 31 December 2016, 13,023 blood donations from the University Hospital Aachen in Germany were routinely screened for West Nile virus (WNV) RNA using the cobas TaqScreen WNV Test. On 28 September 2016, one blood donor was tested positive. Subsequent analysis revealed an acute Usutu virus (USUV) infection. During the ongoing USUV epizootics in Germany, blood transfusion services, public health authorities and clinicians should be aware of increased human USUV infections.

During July–October 2016, several western European countries reported the largest Usutu virus (USUV) epizootic registered so far in Europe causing a massive bird die-off [1]. Blood donor samples collected between 1 June and 31 December in the Institute for Transfusion Medicine, University Hospital, Aachen, are routinely screened for West Nile virus (WNV) RNA. On 17 November 2016, the World Health Organization Collaborating Centre (WHO CC) for Arbovirus and Haemorrhagic Fever Reference and Research in Hamburg was informed about a suspected WNV infection in a blood donor from Aachen. Although the sample was tested positive for the presence of WNV RNA, subsequent sequencing and serological investigations revealed an acute USUV infection of the donor. Here we report the first detection of an acute USUV infection of a blood donor from Germany using a cross-reactive WNV screening test and further successful sequencing of a large portion of the genome using deep-sequencing technology.

## Case description

On 26 September 2016, a plasma pool (n = 16) had been detected WNV-positive (Ct: 40.5) using cobas TaqScreen WNV Test (Roche Diagnostics GmbH, Mannheim, Germany) with a sensitivity of 206.4 copies/mL per single donation. In order to detect the positive plasma sample, each sample from the pool was tested individually and the positive sample identified (Ct: 37.5). The blood donor was a German woman in her late 20s, without any travel history outside Germany in the previous 7 months. Furthermore, she had not left the Aachen region at all in the 3 months prior to blood donation. The healthy donor had not experienced any illness or symptoms in the 6 weeks before donation. She reported several mosquito bites before the donation. Blood and urine samples of the donor were sent to the WHO CC in Hamburg for further characterisation. Results of IgG and IgM immunofluorescent assays for WNV, USUV, tick-borne encephalitis virus (TBEV) and Japanese encephalitis virus (JEV) were negative (titres < 1:20) for the first sample collected on 26 September 2016. In contrast, IgG and IgM seroconversion was demonstrated with the follow up sample collected on 20 November 2016, 55 days later and the results for WNV-IgG (1:160), WNV-IgM (1:160), TBEV-IgG (< 1:20), TBEV-IgM (< 1:20), JEV-IgG (1:640), and JEV-IgM (1:80) and USUV-IgG (1:1280) and USUV-IgM (1:640) suggested a recent USUV infection. The blood donor reported no history of vaccination against YFV and JEV. Extracted RNA of plasma and urine samples were tested for the presence of flavivirus RNA with pan-flavivirus RT-PCR [2]. A positive PCR result was obtained with RNA from the plasma sample and direct Sanger sequencing of the PCR amplicon showed USUV nucleic acid sequence. Attempts to isolate USUV in cell culture using the donor plasma were not successful.

## Deep sequencing and genetic analysis

**Figure 1:**
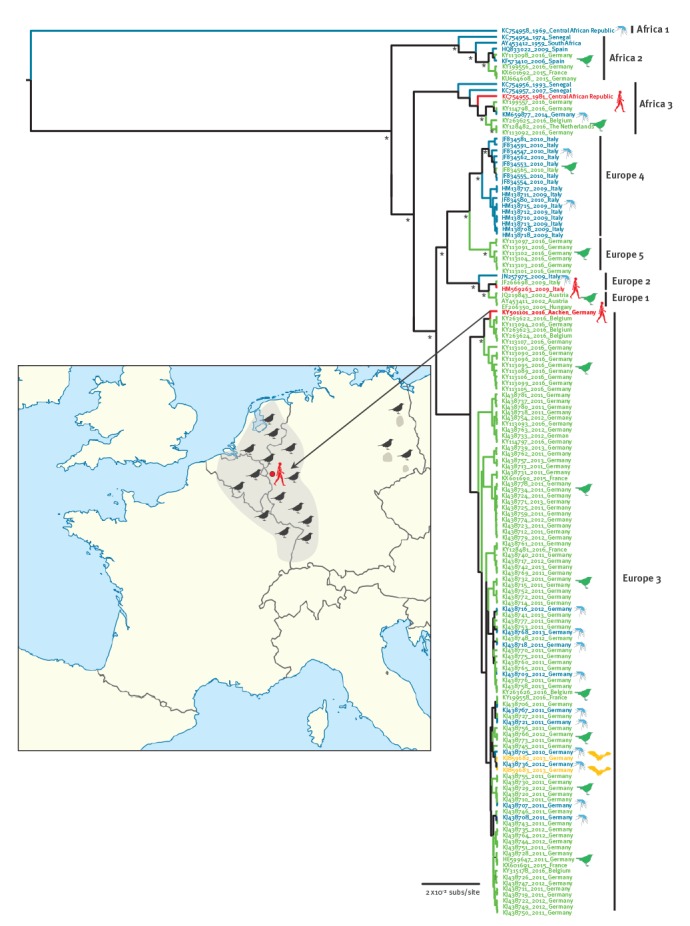
Bayesian maximum clade credibility tree representing the phylogenetic placement of the human Usutu virus (USUV) strain Aachen compared with all available USUV based on partial NS5 gene nt sequences

The concentrated and purified RNA was further subjected to deep-sequencing using in-house next-generation sequencing pipeline in order to obtain larger fragments of the USUV genome. Thereby, we were able to successfully recover about 60% of the USUV polyprotein gene. USUV from the donor plasma showed 99% homology with those found in the birds during the 2016 epizootics corresponding with the same region from where the donor originated (Figure 1). Phylogenetic analysis demonstrated that USUV ‘Aachen’ strain clustered together with the 2016 outbreaks strains and formed together with some German and Belgian strains a distinct subclade within the previously assigned European lineage 3 (Figure 1).

Phylogenetic analysis was performed by using Bayesian Markov chain Monte Carlo (MCMC) tree-sampling method implemented in BEAST v.1.8.0 (http://beast.bio.ed.ac.uk). Statistical supports of grouping from Bayesian posterior probabilities (clade credibilities ≥ 90%) are indicated at the nodes (asterisks). The map indicates the regions of the European countries which have reported USUV outbreaks in 2016 (grey), and the origin of the human USUV Aachen strain. GenBank accession numbers, years of detection and countries of origin for sequences used to construct the tree are indicated on the branches. Scale bar indicates mean number of nt substitutions per site.

**Figure 2:**
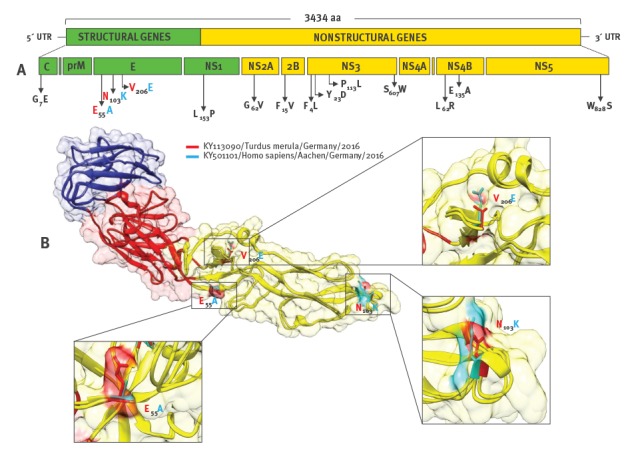
Amino acid mutations in the Usutu virus (USUV) Aachen strain: **A**. schematic representation of the genome organisation of USUV, **B.** structural location of the USUV non-synonymous mutations in the Aachen strain depicted on the predicted E glycoprotein structure

The analysis of the polyprotein gene revealed several host-specific unique amino acid mutations from which three were located in domain II of the envelope glycoprotein (Figure 2).

In Panel A, the numbers indicate the positions and the single letter the unique non-synonymous amino acid mutations of the Aachen strain. Amino acid substitutions in the envelope glycoprotein are magnified and indicated in red and light blue (Aachen strain) respectively.

The three-dimensional ribbon structure of a single monomer of the USUV envelope glycoprotein is shown with the corresponding three viral domains (domain I in red; domain II in yellow; domain III in blue) and surface exposed variable residues magnified. Homology models for USUV envelope protein was constructed using the initial homology search and template selection method in Chimera [18]. The template sequences used to create the USUV E protein model was the crystal structure of the West Nile virus envelope glycoprotein (PDB 2I69). The final 3D structures were prepared and visualised with Chimera v1.11 [18].

## Background

USUV, an Old World flavivirus included in the JEV antigenic complex is transmitted by mosquitoes to birds that act as the main amplifying hosts, while humans are considered incidental or dead-end hosts [3]. Since the first emergence in the mid-1990s in Europe, USUV has been responsible for smaller periodic epizootics in several European countries, the largest one being registered in 2016 [1,4-6]. USUV can cause Usutu fever in humans with mild to severe symptoms characterised by fever, rash, jaundice, headache, nuchal rigidity, hand tremor, and hyperreflexia [7-10]. So far, humans were considered incidental hosts with very low prevalence, but recent data from Italy indicated that human USUV infection may not be a sporadic event and is more frequent than WNV infections [11]. In 2012, 1 of 4,200 blood donors from south-west Germany was tested positive for USUV-specific IgG and IgM antibodies demonstrating a recent USUV infection of the donor [12]. However, there is no documented case of Usutu fever caused by transfusion of USUV-contaminated blood products.

## Discussion and conclusion

The present report, including serological and molecular findings, suggests an acute and asymptomatic USUV infection of a blood donor in Germany in late summer of 2016. The Bayesian phylogenetic analysis revealed that the USUV sequence of the blood donor had a high sequence homology with recent strains responsible for the 2016 USUV epizootics in the western part of Germany from where the donor lived. Since the blood donor had no history of travelling abroad in the 7 months before the end of September 2016, she must have been infected in Germany, which, together with the genetic data obtained, further strengthens an autochthonous USUV infection in the Aachen region.

USUV is considered an emerging arbovirus due to its rising incidence of human infections that are likely to be frequent as WNV infections and the expansion in new, previously known USUV-free areas [1,11]. It is interesting to note the amino acid mutations detected mostly in the envelope protein and NS5 gene. Although the biological consequences of these mutations are not known, similar changes in the related WNV increased the sensitivity to neutralisation by a monoclonal antibody targeting a cryptic epitope in the fusion loop and altered tropism and neuroinvasive capacity [13,14]. The detection of USUV RNA in the blood donor sample using cobas TaqScreen WNV Test, demonstrates the capability of this test to detect other flaviviruses than WNV due to cross-reactivity of the used primer-probe reagents.

To address the emergence of WNV regarding blood safety, the Federal Institute for Vaccines and Biomedicines (Paul-Ehrlich-Institut) as the responsible authority in Germany, implemented a regulation for non-pathogen inactivated blood components in 2003, last updated in 2014 [15]. Since the update in 2014, alternatively to the deferral period of 28 days, donor eligibility is accepted indicating a non-reactive screening result using a nucleic acid amplification technique (NAT)-based test for WNV RNA with a minimum detection sensitivity of 250 copies/mL for each donor sample [15].

Recent molecular and serologic surveillance studies in Germany and neighbouring countries identified epizootic hotspots for USUV that could help to initiate targeted vector control programs to prevent human exposure to the virus [1,3,16,17]. Moreover, the present report highlights the potential risk of transfusion-associated transmission of USUV. However, until now there is no reported case of transfusion-associated Usutu fever in Europe. The demonstrated case should raise awareness of the risk of USUV infection in humans during epizootics, especially in late summer.

## References

[1] Cadar D Lühken R van der Jeugd H Garigliany M Ziegler U Keller M Widespread activity of multiple lineages of Usutu virus, western Europe, 2016. Euro Surveill. 2017;22(4):30452. 10.2807/1560-7917.ES.2017.22.4.30452 28181903 PMC5388094

[2] Becker N Jöst H Ziegler U Eiden M Höper D Emmerich P Epizootic emergence of Usutu virus in wild and captive birds in Germany. PLoS One. 2012;7(2):e32604. 10.1371/journal.pone.0032604 22389712 PMC3289667

[3] Engel D Jöst H Wink M Börstler J Bosch S Garigliany MM Reconstruction of the Evolutionary History and Dispersal of Usutu Virus, a Neglected Emerging Arbovirus in Europe and Africa. MBio. 2016;7(1):e01938-15. 10.1128/mBio.01938-15 26838717 PMC4742707

[4] Rijks JM Kik ML Slaterus R Foppen R Stroo A IJzer J Widespread Usutu virus outbreak in birds in the Netherlands, 2016. Euro Surveill. 2016;21(45):30391. 10.2807/1560-7917.ES.2016.21.45.30391 27918257 PMC5144937

[5] Weissenböck H Kolodziejek J Url A Lussy H Rebel-Bauder B Nowotny N Emergence of Usutu virus, an African mosquito-borne flavivirus of the Japanese encephalitis virus group, central Europe. Emerg Infect Dis. 2002;8(7):652-6. 10.3201/eid0807.020094 12095429 PMC2730324

[6] Weissenböck H Bakonyi T Rossi G Mani P Nowotny N Usutu virus, Italy, 1996. Emerg Infect Dis. 2013;19(2):274-7. 10.3201/eid1902.121191 23347844 PMC3559058

[7] Cavrini F Gaibani P Longo G Pierro AM Rossini G Bonilauri P Usutu virus infection in a patient who underwent orthotropic liver transplantation, Italy, August-September 2009. Euro Surveill. 2009;14(50):19448. 20070935

[8] Pecorari M Longo G Gennari W Grottola A Sabbatini A Tagliazucchi S First human case of Usutu virus neuroinvasive infection, Italy, August-September 2009. Euro Surveill. 2009;14(50):19446. 20070936

[9] Vilibic-Cavlek T Kaic B Barbic L Pem-Novosel I Slavic-Vrzic V Lesnikar V First evidence of simultaneous occurrence of West Nile virus and Usutu virus neuroinvasive disease in humans in Croatia during the 2013 outbreak. Infection. 2014;42(4):689-95. 10.1007/s15010-014-0625-1 24793998

[10] Santini M Vilibic-Cavlek T Barsic B Barbic L Savic V Stevanovic V First cases of human Usutu virus neuroinvasive infection in Croatia, August-September 2013: clinical and laboratory features. J Neurovirol. 2015;21(1):92-7. 10.1007/s13365-014-0300-4 25361698

[11] Grottola A Marcacci M Tagliazucchi S Gennari W Di Gennaro A Orsini M Usutu virus infections in humans: a retrospective analysis in the municipality of Modena, Italy. Clin Microbiol Infect. 2017;23(1):33-7. 10.1016/j.cmi.2016.09.019 27677699

[12] Allering L Jöst H Emmerich P Günther S Lattwein E Schmidt M Detection of Usutu virus infection in a healthy blood donor from south-west Germany, 2012. Euro Surveill. 2012;17(50):20341. 23241231

[13] Gaibani P Cavrini F Gould EA Rossini G Pierro A Landini MP Comparative genomic and phylogenetic analysis of the first Usutu virus isolate from a human patient presenting with neurological symptoms. PLoS One. 2017;23(1):33-7. 10.1016/j.cmi.2016.09.019 23741387 PMC3669420

[14] Goo L VanBlargan LA Dowd KA Diamond MS Pierson TC A single mutation in the envelope protein modulates flavivirus antigenicity, stability, and pathogenesis. PLoS Pathog. 2017;13(2):e1006178. 10.1371/journal.ppat.1006178 28207910 PMC5312798

[15] Paul-Ehrlich-Institut. Federal Institute for Vaccines and Biomedicines. Änderung des Bescheides über die Anordnung des Ausschlusses von Blutspendern zur Verhinderung einer möglichen Übertragung des West-Nil-Virus durch nicht pathogen-inaktivierte Blutkomponenten. [Public notification on implementation of risk minimisation measures for blood components concerning transmission of West Nile Virus]. 22 Jan 2014. German. Available from: https://www.pei.de/SharedDocs/Downloads/vigilanz/haemovigilanz/bescheide/2014-04-11-anordnung-ausschluss-blutspender-wnv-ergaenzung.pdf?__blob=publicationFile&v=6

[16] Garigliany M Linden A Gilliau G Levy E Sarlet M Franssen M Usutu virus, Belgium, 2016. Infect Genet Evol. 2017;48:116-9. 10.1016/j.meegid.2016.12.023 28017913

[17] Lecollinet S Blanchard Y Manson C Lowenski S Laloy E Quenault H Dual Emergence of Usutu Virus in Common Blackbirds, Eastern France, 2015. Emerg Infect Dis. 2016;22(12):2225. 10.3201/eid2212.161272 27869608 PMC5189168

[18] Pettersen EF Goddard TD Huang CC Couch GS Greenblatt DM Meng EC UCSF Chimera--a visualization system for exploratory research and analysis. J Comput Chem. 2004;25(13):1605-12. 10.1002/jcc.20084 15264254

